# “One More Picture”: Caption Content and Empathy Gains in a Photo Captioning Intervention for Dementia-Caregivers

**DOI:** 10.1093/geroni/igaf122.287

**Published:** 2025-12-31

**Authors:** Emily Mroz, Rarinsri Assakul, Jenna Wells, Molly Perkins, Kenneth Hepburn, James Rilling

**Affiliations:** Emory University, Atlanta, Georgia, United States; Cornell University, Ithaca, New York, United States; Cornell University, Ithaca, New York, United States; Emory University School of Medicine, Atlanta, Georgia, United States; Emory University, Atlanta, Georgia, United States; Emory University, Atlanta, Georgia, United States

## Abstract

Empathic perspective-taking allows caregivers to better understand, communicate with, and respond to the needs of family members living with dementia. A photo captioning intervention can promote empathic perspective-taking by guiding caregivers to take daily photographs of their family member living with dementia and caption those photos to capture the inner voice of that care recipient. In this study, we analyzed patterns in 190 photo captions from a pilot trial of this intervention with dementia-caregivers. We used a subgroup parallel sampling design which allowed us to identify patterns in photo captions for the participants who experienced the most and least change in perspective-taking across the intervention. Descriptive content-analysis demonstrated that caregivers who experienced high perspective-taking change wrote photo captions that 1) maintained first-person, care-recipient focused language, 2) highlighted care recipients’ engagement and intention, and 3) illustrated resilient living. Caregivers who experienced low perspective-taking change captioned photos by 1) offering inconsistent, incomplete phrasing, 2) providing generic, detected descriptions, and sometimes 3) adopting a negative, critical tone. Caregivers from both groups also wrote captions that 1) connected care recipients’ emotions to events and 2) recorded care recipients’ wonders, wants, and wishes. The ways that caregivers approach writing photo caption content may be an important behavioral mechanism influencing the impact of this intervention. Results can guide refinement of perspective-taking interventions to maximize benefits experienced by caregivers and their care recipients.

